# Making Mobile Nanotechnology Accessible: Is the Explicit Preparation of Janus Nanoparticle Necessary to Achieve Mobility?

**DOI:** 10.3390/nano14221796

**Published:** 2024-11-08

**Authors:** Vagisha Nidhi, Arthur Allaire, Zakariya Ait Athmane, Patrick Guenoun, Fabienne Testard, Jean-Philippe Renault, Florent Malloggi

**Affiliations:** Université Paris-Saclay, CEA Saclay, CNRS, NIMBE, UMR 3685, LIONS, 91191 Gif-Sur-Yvette CEDEX, Francearthur.allaire@ens.psl.eu (A.A.); patrick.guenoun@cea.fr (P.G.)

**Keywords:** self-propulsion, mobility, chemotaxism, Janus particles, gold–silica particles, DLS, PTV

## Abstract

This study compares the mobility behaviour, in a H_2_O_2_ environment, of three different geometries of hybrid particle made of silica core functionalized by gold (nanoparticles or layer). It is known that the decomposition of H_2_O_2_ on gold surfaces drives mobility; however, the link between mobility orientation and the organization of gold on silica surfaces is still questionable. While conventional wisdom posits that asymmetric designs are crucial for generating phoretic forces or localized bubble propulsion, recent research suggests that symmetrical particles may also exhibit motility. To address this debate, we developed a robust workflow for synthesizing gold grafted silica nanoparticles with precise control over size and shape, enabling the direct comparison of their motile behaviour by dynamic light scattering and particle tracking velocimetry. Our results indicate, first, that a combination of techniques is necessary to overcome their intrinsic limitation and, second, that the inherent asymmetry generated by isotropic gold nanoparticle deposition onto silica surfaces may enable particle motility.

## 1. Introduction

Nano- and microswimmers, nano- and microscale devices that are capable of converting various forms of energy into mechanical motion, have garnered significant attention due to their potential applications in targeted drug delivery, nanoscale assembly, environmental sensing, and remediation [1]. For more than a decade, different strategies have been used to develop artificial materials inspired by self-propulsed microorganisms or bacteria [2]. Various forms of energy input—chemical, optical, magnetic, or electrical—were used to drive nano- or micromotors. Optical propulsion, for example, exploits light-induced forces, such as those arising from plasmonic effects, to achieve movement [3]. While, chemical propulsion usually involves catalytic reactions on the nanomotor’s surface [2]. In such case, a fuel source is generally present around the nanomotors. It is currently thought that, if the particle is asymmetric, these catalytic reactions create chemical and/or thermal gradients that generate propulsion through phoretic forces [4,5]. For a specific fuel like H_2_O_2_ that decomposes with gas production, an alternative mechanism is localized bubble propulsion [6]. These considerations have led to the design of nanoswimmers with pronounced asymmetries [7,8,9,10]. Thus, Janus particles (made of two different faces) [11] are generally designed to confer asymmetry to isotropic spherical shapes. The requirement of asymmetry is so deeply rooted that, in most of these studies, the non-asymmetric particles were not even compared to the asymmetric ones. However, some others have argued that a perfect asymmetry is not mandatory to obtain motion [12,13,14]. When a taxis is sought, Janus structure may even be detrimental as it causes poor directionality due to reorientation of the catalytic side. Propulsion then relies on the self-generated concentration field around the colloid [15,16]. It appears that fast asymmetric catalysis does not necessarily provide efficient self-propulsion beyond the Brownian motion, even though a strict comparison of Janus and isotropic particles is scarcely described [17]. All these observations question the mechanisms of self-propulsion (self-electrophoresis, self-diffusion, etc…) and the requirement to design optimized nanomotors. As, obviously, Janus particles are quite complex to prepare with reproducibility and geometry control, it would be of tremendous interest to obtain mobile particles without particular asymmetry.

In this paper, we designed a robust particle preparation workflow allowing to compare exactly the mobility of particles made of similar composition except for their geometry and asymmetry. The synthetic pathways were chosen to prepare a large amount of particles in a reproducible manner. For such a precise control, gold was preferred to platinum or to catalase, as its nanoparticle preparation is much more controlled and its lower activity minimize bubble production [18]. The changes in diffusion were measured in two conditions, (i) in bulk using DLS, and (ii) in a microfluidic channel, in order to suppress collective effects.

## 2. Materials and Methods

### 2.1. Materials

The materials used in this study, (3-Aminopropyl) triethoxysilane (APTES) 99% (CAS N°: 919-30-2), Hexane (CAS N°: 110-54-3), Paraffin wax (CAS N°: 8002-74-2), Poly-L-lysine (PLL) solution 0.1%_w/v_ in H_2_O (CAS N°:25988-63-0), Poly(allylamine hydrochloride) (PAH) M_w_ ~17,500 (CAS N°:71550-12-4), Tetraethyl orthosilicate (TEOS) 99.99% (CAS N°: 78-10-4), Sodium chloride (NaCl) (CAS N°:7647-14-5), Sodium citrate tribasic dehydrate (CAS N°: 6132-04-3), Hydrogen Peroxide (30%_v/v_) (CAS N°: 7722-84-1) and Polydimethylsiloxane (PDMS) Sylgard® 184, were purchased from Sigma Aldrich (Saint Quentin FallavierFrance). Cetyltrimethylammonium bromide (CTAB 98%) (CAS N°:57-09-0) was purchased from G BIOSCIENCES (St. Louis, MO, USA), and Citric acid monohydrate (CAS N°: 5949-29-1) from Fluka from Fisher-Scientific SAS (Illkirch, France). Ammonia 28% and Ethanol Absolute were purchased from VWR Chemicals (Fontenay-sous-Bois, France), Whatman 1 filter paper from Cytiva from VWR (Fontenay-sous-Bois, France) (Ø 90 mm, Cat. 1; CAS N°: 1001-090). All the chemicals purchased were of analytical grade and used without further purification. Samples were prepared with Milli-Q^®^ water.

### 2.2. Synthesis of Gold and Silica Nanoparticles (AuNPs and SNP Respectively)

These classical syntheses are described in the Supporting Information Parts S1 and S2, Figures S1, S2 and S4.

### 2.3. Synthesis of Isotropic Gold/Silica Particles (ISO)

Isotropic gold/silica particles (called isotrope or ISO) are synthesized in two steps. First, a soft APTES functionalization of the particles surface in water is adapted from the Bottero et al. protocol [19]; second, the AuNPs are attached on the functionalized particles. For APTES functionalization, the silica nanoparticles (SNPs) stock suspension is pre-redispersed by 10 min in ultrasonic bath (at 37 Hz, 60% power). These conditions did not lead to particle degradation (see Figure S3). Then, in a Teflon flask, 0.5 mL of the prepared SNP suspension is added to 50 mL of ethanol (7%_v/v_)/water mixture), mixed in a vortex for 30 s, followed by the addition of 1 mL APTES (in excess to be above 15 APTES per nm^2^) [19]. The suspension is left on a rotatory agitator for 18 h at 50 rpm. Finally, excess APTES is removed through centrifugation/washing cycles, with centrifugation at ~11,000 RCF (7000 RPM) for 15 min, two ethanol washing cycles, the final one in water. The obtained (clean) SNP–NH_2_ are redispersed in 10 mL water and stored in the fridge at 6 °C for further usage. For AuNPs attachment, the SNP–NH_2_ are first redispersed with an ultrasonic bath, then 5 mL of the SNP–NH_2_ suspension is kept under an ultrasonic bath for 1–2 mn (at 37 Hz, 60% power) while 1 mL of the AuNPs suspension is added slowly. The resulting suspension is placed on a rotatory agitator at 50 RPM for 10 h. Excessive AuNPs are removed by centrifuging at ~1400 RCF (2500 RPM) for 15 min and the pellet is resuspended in 5 mL of water. The ISO particles are characterized with SEM and EDX. Prior to their use, the suspension needs to be under an ultrasonic bath and vortex mixed.

### 2.4. Gold/Silica Janus Nanoparticles (JP) with Chemical Synthesis

**Figure 1:**
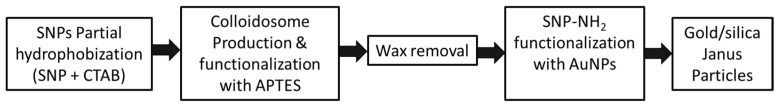
Summary flowchart of the chemical synthesis protocol for gold/silica Janus Particle via the Pickering emulsion method.

Figure 1 describes the protocol for Janus particle synthesis. First, wax colloidosomes stabilized by SNPs are produced (from a Pickering emulsion template), then functionalized with APTES to produce SNP–NH_2_ Janus particles. In a final stage, the wax is removed and AuNPs are attached following the same protocol as for isotropic gold/silica particles.

For Janus synthesis, the experimental parameters (CTAB concentration, silica concentration, ultraturax energy, APTES concentration, AuNPs concentration, ultrasonic mediation, etc…) were optimized to ensure the reproducibility of the Janus particle production from one batch to another.

### 2.5. Synthesis of Colloidosomes

The colloidosomes are synthesized following mainly Avossa et al. [20] procedure and former publications [21,22,23]. In a 25 mL beaker, 20 mL of water and 2.5 mL of SNP solution (to attain the same total SNP surface as Avossa et al. [20]) were mixed. The suspension is left under an ultrasonic bath for 5 to 10 min (at 37 Hz, 60% power). Then, a chosen volume of cetyltrimethylammonium bromide (CTAB) from a stock solution (C = 0.2 mM) is added to change the wetting properties of SNPs, and the mixture is stirred magnetically for 30 min at 50 °C (1000 RPM). Several concentrations of CTAB (from 0.005 mM to 0.08 mM) were tested to optimize the protocol, and the optimized final CTAB concentration is 0.04 mM. In a second step, the suspension is heated at 75 °C and a pre-melted amount of wax corresponding to 0.8/10 in weight of water is added under ultraturax mixing. After 2 min at a power of 9500 RPM, the emulsion is quenched in 100 mL of cold water. Then, the suspension is filtered on a Buchner funnel with a Whatman filter (Cat. 1; CAS N°: 1001-090) and rinsed with ≈150 mL of water. Finally, the solid is recovered and left to dry at room temperature. The final product is characterized with SEM on roughly 60 colloidosomes measured.

### 2.6. Synthesis of Janus SNP–NH_2_ Particles

We use the same soft protocol in water as for isotrope particles, derived from Bottero et al. to graft APTES on the silica embedded in the wax. An amount of 0.5 g of colloidosomes is dispersed in a total volume of 50 mL of EtOH (7%_v/v_)/H_2_O mixture in a Teflon flask followed by the addition of 0.2 mL APTES (in excess to be above 15 APTES per nm^2^). The suspension is left on a rotatory agitator at 50 rpm for 18 h. Then, the suspension is filtered on a Buchner funnel with a Whatman filter (Cat. 1; CAS N°: 1001-090) and washed with ethanol to remove the excess APTES. To recover the functionalized Janus SNP–NH_2_ nanoparticles, the wax is removed with hexane. The powder is added to ≈ 40 mL of hexane in a polypropylene centrifuge tube (Vt = 50 mL), left in an ultrasonic bath (37 kHz) for 30 min and vortexed right after. The NPs are first extracted and washed from four centrifugation (7000 RPM for 15 min)/washing cycles (two cycles with hexane, one with ethanol, and one with water). Finally, the Janus SNP–NH_2_ particles are concentrated in ≈10 mL of water. The particles were stored in the fridge and characterized by DLS, IR, and SEM.

### 2.7. Synthesis of Janus SNP–AuNP Particles (JP)

To partially functionalize silica particles with gold, the same protocol as for isotrope SNPs has been used on the Janus SNP–NH_2_ particles. An amount of 5 mL of the SNP–NH_2_ suspension is placed in a Falcon tube under sonication while 1 mL of the gold suspension is added slowly. The suspension is left for 1–2 min before adding another 1 mL of AuNP suspension to reach a total volume of 5 mL added in the end. Finally, the Falcon tube is left on the rotatory agitator at 50 RPM for 10 h. To remove excess AuNPs, the suspension is centrifuged at 2500 RPM for 15 min. This procedure is repeated at least three times (until an uncoloured supernatant is obtained) before concentrating the Janus particles in 5 mL of water. The Janus particles produced were then stored in the fridge before further characterization (SEM, EDX, UV, etc...).

### 2.8. Synthesis of Janus Gold/Silica Nanoparticles with Physical Protocol (PVD)

To produce Janus particles made of a thin gold layer partially covering the silica particles, a physical vapour deposition method was used. An amount of 200 µL of the silica particle suspension is first deposited by spin coating on a glass slide at 500 RPM for 10 s, followed by 3400 RPM for 30 s. The glass slide was pre-cleaned under plasma treatment with oxygen (1 min plasma under 50 W power and 7.86 e^−1^ torr pressure). The obtained SNPs glass coated slide was let in into a physical vapour deposition device at 10^−6^ mbar pressure and 100–140 A current running in the crucible to deposit a 0.5 nm thick chromium layer at the rate of 0.5 angstrom/s, followed by a 20 nm thick gold layer deposition from top at 1.6 to 1.9 angstrom/s deposition [24,25]. To recover the Janus particles, the glass slide was deposited into 10 mL water in a petri dish placed under an ultrasonic bath for 30 min (37 Hz, 100%) [26]. The diluted suspension of Janus particles collected into a polypropylene centrifuge tube are centrifuged for 15 min ~8064 RCF (6000 RPM). The pellet is redispersed in 1 mL of Milli-Q^®^ water and stored in the fridge. The particles are characterized by SEM.

### 2.9. Characterization Techniques

#### 2.9.1. Scanning Electron Microscopy (SEM)

A ZEISS Ultra 55 equipped with a field emission gun (FEG), coupled with a Bruker EDX (Energy Dispersive X-ray Spectrometry) was utilized for SEM analysis of the particles. An amount of 1.5 kV and 10 kV EHT (Electron High Tension) was maintained during characterizations. Either carbon tape or silicon wafer (surface cleaned via UV-ozone cleaner for 45 s) [27] were used as sample substrate. One µL of particle suspension (after 15 min ultrasonic bath) was dried on sample substrate during sample preparation for each SEM observations.

#### 2.9.2. Dynamic Light Scattering (DLS), Measurement

A Zetasizer Nano Series Nano from Malvern Panalytical (Laser wavelength 633 nm) was utilized for DLS measurements using the configuration of backscattered angle of 173° (NIBS default), 25 °C temperature, and equilibration time of 2 min. The measurements proceeded after a dilution step in Milli-Q^®^ water (refractive index (1.33) and viscosity (0.89 mPa.s)). The particle concentration was adapted for each category to have an optimized signal (mean count rates of scattered lights for optimal dilution was maintained from 160 to 480 kcps). The experiments were realized by preparing fresh samples before each DLS observation. To mitigate the risk of dust and contamination, a new plastic container was used for each experiment, and before using new containers, they were rinsed thrice (with milli-Q^®^ water). This precautionary measure was followed to ensure the integrity of the experimental setup and minimize the impact of contaminants on DLS measurements [28,29]. First, correlograms are registered with three repeated experiment measurements including three analyses for each. Then, size distribution is extracted using the methods of cumulants for AuNPs and SNPs [30]. For the other particles, a particular treatment has been used (see Section 2.10).

For the case of measurements in the presence of H_2_O_2_, particles were first submitted to ultrasonic bath for 5 min and vortexed for 10 s, then added to Milli-Q^®^ water/H_2_O_2_ mixture pre-mixed with a glass tip for 10 s (to avoid metal contamination to which H_2_O_2_ is very sensitive). The concentrations of H_2_O_2_ were 0%_v/v_ H_2_O_2_, 0.5%_v/v_ H_2_O_2_, 1%_v/v_ H_2_O_2_ and 5%_v/v_ H_2_O_2_ for SNP; Iso, JP, and PVD.

### 2.10. Dynamic Light Scattering (DLS), Data Treatment

Correlogram; autocorrelation function (C), is a function of lag-time (∆t) and can be described by Equation (1) where A is the scattering amplitude (normalized factor), B the instrumental factor, q the scattering vector (Equation (2)), and D the diffusion coefficient. The diffusion coefficient can be calculated from DLS correlogram fitting using Equation (1) plus (2) [31,32]. The scattering vector q for the set-up is 2.62 × 10^7^ m^−1^ (calculated from Equation (2) where n is refractive index (1.33), θ is detector angle detector (173°), and λ is wavelength (633 nm)). The theoretical values of diffusion coefficient for translational diffusion can be calculated from the Stokes–Einstein equation (Equation (3)) [33], and linked to the particles diameter, where k_B_ is the Boltzmann constant (1.380649 × 10^−23^ m^2^kg/(K·s^2^)), T is Temperature (298 K), μ is the dispersant-viscosity (0.00089 kg/(m·s)) [34] and R the hydrodynamic radius of the particle (m).

(1) $$C\;\left(\Delta t\right)=A\;e^{\left(-2Dq^{2}∆t\right)}+B$$

(2) $$q=\frac{4\pi n}{\lambda }\left(\sin\frac{\theta }{2}\right)$$

(3) $$D=\frac{k_{B}T}{6\pi \mu R}$$

The particles are also subject to rotational diffusion with a characteristic timescale τ_R_^−1^ = k_B_T/(8πμR^3^).

Hence for anisotropic object, the decay of the correlation function contains the superposition of translational and rotational motion [35]. These two contributions could only be separated from depolarized dynamical scattering.

If fitting by one exponential decay is too poor, a double exponential decay is used. This happens for the Janus, ISO, and PVD particles as shown in the results below.

### 2.11. Experiment: Particle Tracking Velocimetry (PTV)

PTV analyses were obtained in a channel included in a PDMS (Polydimethylsiloxane) microfluidic chip (~45 µm and 180 µm channel height and width, respectively) made by soft lithography protocol [36]. Prior to the measurements, particle suspension is filtered (pore size 0.65 µm) and diluted in Milli-Q^®^ water (1:2 for SNP, 1:1 for ISO and JP) to eliminate any particle-aggregates that could have formed due to aging of samples. Particles/water/H_2_O_2_ suspensions are prepared just before the analysis by mixing 0, 17, and 33 μL H_2_O_2_ with 100, 83, and 67 μL particle suspension to attain 0%_v/v_, 5%_v/v_, and 10%_v/v_ H_2_O_2_, respectively_._ The obtained suspensions are mixed manually for 10 s with micropipette prior to analysis. Then, samples are flowed in the chip by bringing pressure at 1 mbar. The flow is stopped after 1 min to avoid convection driven motion in particle and measurements of particle-motion were performed after 2 min stabilization time at ambient pressure. Conditions for imaging are 10 ms of camera exposure, frame rate at 10 frames per second (FPS), and record 200 images at 40× magnification. The intensity of the microscope-lamp has to be minimized to limit light-induced decomposition of hydrogen peroxide and motion from thermal effects. The experimental protocol was validated on the pristine silica particles.

### 2.12. Data Treatment (PTV), Image Processing (TrackMate)

The PTV study is in 2D, and sticking of particle on the surface of glass slide is inevitable; therefore, imaging in the region of interest (ROI) was chosen away from channel boundaries. After preliminary adjustments (image brightness, contrast) images were observed through TrackMate-ImageJ for tracking particle trajectories [37]. For this purpose, pixel width, height, and voxel depth were calibrated to unit value. Further, LoG (Laplacian or Gaussian) detector, object diameter, quality threshold, tracker was set followed by the optimal criteria settings of the tracker type. In this PTV apparatus, a simple LAP (linear assignment problem) tracker was utilized to benefit in gap-closing between spots, linking-distance; beneficial for our 2D set-up with particles appearing and disappearing within close proximity. Lastly, information like particle-track, speed, travelled-distance, etc. (obtained as .csv output) were processed on Python platform PyCharm.

As the frame rate (10 FPS), exposure time (10 ms), and pixels per μm (6.67) were known values, speed obtained from TrackMate (Track Mean Speed) was converted from ‘pixels per frame’ to ‘μm/s’ using Equations (4)–(6), followed by calculation of mean diffusion coefficient using Equation (7) and the mean square displacement (MSD) [38,39] derivable from Velocity^2^ × Time^2^. Comparing with Equation (7), Equation (8) is obtained, with n the number of dimensions (2), D the diffusion coefficient and T_d_ the Time interval per frame (0.09 s) [40].

(4) $$Time\;per\;frame=\frac{1}{frame\;rate}$$

(5) $$T_{d}=Time\;per\;frame-Exposure\;time$$

(6) $$v\left(µm/s\right)=Speed\left(px/frame\right)\times \frac{{pixel}_{size}}{T_{d}}$$

(7) $$D=\frac{v^{2}T_{d}}{2n}$$

(8) $$MSD=2nDT_{d}$$

### 2.13. Data Treatment (PTV), Data Processing (Python)

**Figure 2:**
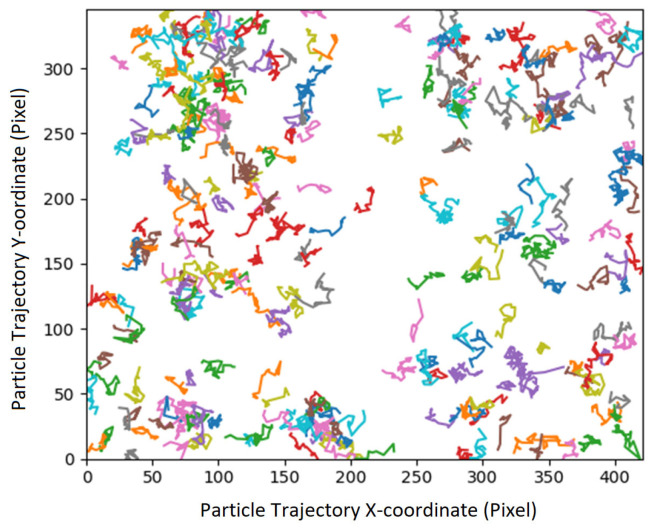
Trajectories of SNP in 0%_v/v_ H_2_O_2_ from Tracking as given by TrackMate V7.10.

Particle trajectories (Figure 2) (obtained from TrackMate-Spot Results) were repositioned to origin axis (zero, zero Cartesian coordinates) by fetching the respective x and y coordinates and combining it with its respective displacement-paths for analysis. For example, Figure 2 shows native trajectories for observed SNP (in 0%_v/v_ [H_2_O_2_]).

## 3. Result and Discussion

### 3.1. Particle Preparation

**Figure 3:**
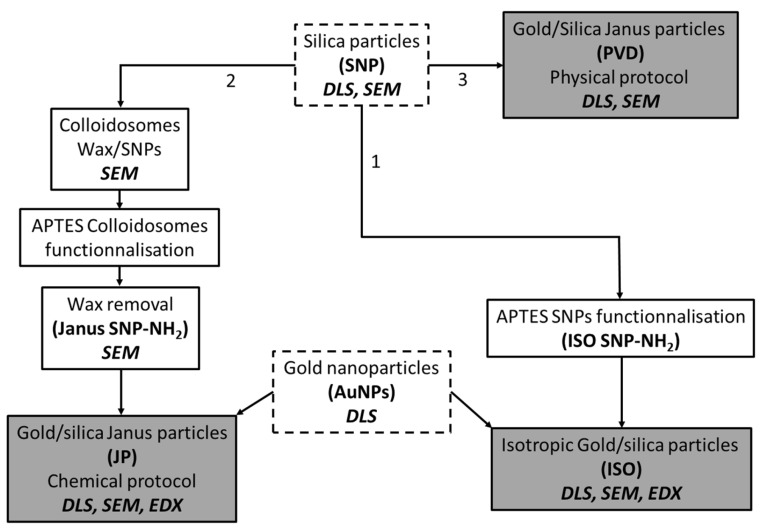
Flowchart of the three strategies used to produce Janus and isotropic particles. 1: ISO, 2: JP through Pickering emulsion and colloidosomes method, 3: PVD. The characterisation methods are indicated in bold and italic. The precursors particles SNP and AuNPs are indicated surrounded with dashed line.

Silica particles half covered by gold (Janus particles) and isotropic particles (fully covered by gold) were formulated to compare their properties. The particles are produced through attachment of gold nanoparticles (AuNPs) or from the deposition of a thin layer of gold on silica particles. Four classes of particles were synthesized: pristine silica (called “SNP”), isotropic AuNPs/Silica particles (called “ISO”), Janus AuNPs/Silica particles (called “JP”) and Janus thin Au layer/Silica particles (called “PVD”) (see Figure 3 for the scheme of the different synthetic pathways). The silica particles were classically synthesized following the Stöber method [41,42] to produce 475 ± 50 nm size particles (Figures S1 and S2). The AuNPs of ~25 nm stabilized by citrate were synthesized using a reverse Turkevich protocol from Sivaraman et al. [43] (Figure S4).

### 3.2. Gold Nanoparticles Attachment

**Figure 4:**
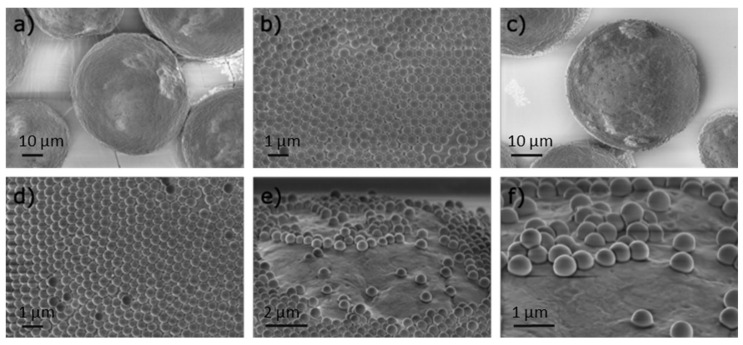
SEM images of the colloidosomes: (**a**,**b**) colloidosome and its surface synthesized at 0.04 mM; (**c**,**d**) colloidosome and its surface synthesized at 0.02 mM; and (**e**,**f**) a closer look on the embedment of silica particles for 0.02 mM CTAB.

For gold nanoparticles attachment, the same chemical procedure is applied for Janus and isotropic particles to ensure similar linkage between gold and silica particles. APTES is first grafted on silica surface in soft conditions (water and room temperature) following a protocol described by Bottero et al. [19] to produce silica particles functionalized with amine group. Then, preformed citrate gold nanoparticles are attached trough the amine function to the silica particles by mixing under sonication the amine grafted silica with the AuNPs water suspension. When applied on isotropic amine grafted particles, isotropic AuNPs/Silica particles are obtained and characterized by SEM. For the Janus particles, a prior step is needed to ensure a partial masking of the silica particles surface prior to the partial grafting by APTES of free silica surface. This can be achieved by the scalable wax Pickering emulsion method developed for 100 to 500 nm silica particles by Ravaine et al. [11,22], and initially proposed by Granick et al. for larger silica particles [21]. It consists of attaching silica particles at the solidified wax/water interface of the so-called “colloidosomes”. Colloidosomes were obtained through the slight hydrophobization of the silica particles with CTAB, to produce an oil in water Pickering emulsion above the melting point of the wax before a temperature quench in ice to solidify the wax. The optimal concentration of CTAB was identified by a combined zeta potential measure of silica coated with CTAB [20] (Figure S5) and SEM analysis of the colloidosomes produced for four CTAB concentrations below the CTAB critical micellar concentration (Figures S6–S8). CTAB concentrations of 0.02 mM and 0.04 mM produced well-defined colloidosomes for the chosen wax (Figure 4) with size varying from ~39 to 55 µm, respectively (Figure S9 and Table S1). The monolayer of partially embedded SNPs in 0.02 and 0.04 mM CTAB (observed at higher magnification in (Figure 4d,b), respectively) confirms SNPs wettability altered via aliphatic chains of CTAB. 

**Figure 5:**
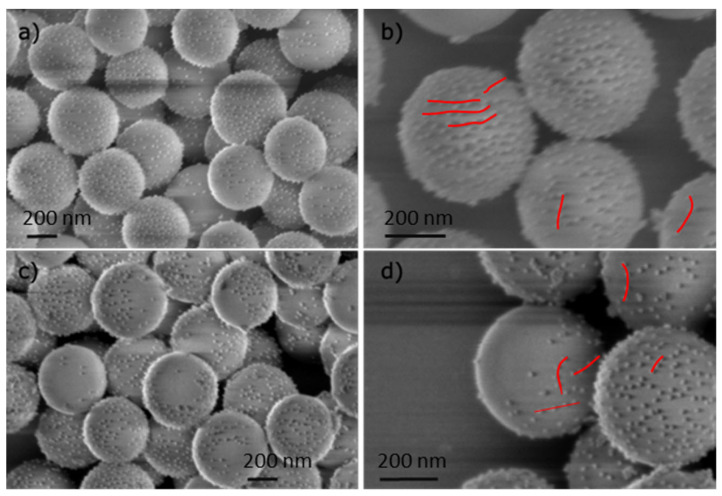
SEM images of (**a**,**b**) isotropic silica nanoparticles functionalized with AuNPs (ISO) and (**c**,**d**) Janus AuNPs/silica nanoparticles synthesized from colloidosomes strategy (JP). The red lines show the particle alignments.

With this approach, the colloidosomes serve as a platform for partial grafting of the silica particles. The surface of silica not embedded in the solidified wax is grafted with APTES in soft conditions similar to the conditions used for isotropic particles. The Janus SNP–NH_2_ particles were then isolated by dissolving wax in hexane (Figure S10). Finally, the Janus SNP–NH_2_ particles were functionalized by AuNPs with the same protocol used for isotropic particles. Figure 5 shows the SEM characterizations of isotropic and Janus particles made of silica particles with grafted AuNPs. The gold and Si identification was verified by EDX for isotropic and Janus particles (Figures S11 and S12, respectively); however, quantification was not possible because of the Si wafer used for the analysis.

### 3.3. Comparison Between JP and ISO, Synthesized with AuNPs Attachment

The silica coverage rate by AuNPs and the average distance between grafted AuNPs can be extracted from the SEM images. Distance between particles can be directly measured on the SEM images or calculated in average from the number of AuNPs per SNP. Table 1 summarizes the obtained values.

The number of gold nanoparticles per SNP, for isotropic and Janus differs by approximately a factor of 2 (see Table 1). The face-to-face distance between AuNPs is slightly inferior to the size of AuNPs diameter for both kind of particles ensuring that the produced particles are similar and only differ by the presence of an area of depleted AuNPs for JP. The minimal distance between the AuNPs results from the equilibrium between VdW interaction and electrostatic repulsion between AuNPs (for 25 nm AuNPs size, the equivalence of the VdW potential with thermal energy is at 20 nm, and for 20 nm AuNPs size, it is 15 nm) [44,45]. However, this minimal distance does not result in a 2D organized paving of the space, but rather as lines of AuNPs that may reveal a subsequent order in the SNPs or in the grafting process. Multiple SEM observations of JP highlighting distinctive asymmetric assembly of gold nanoparticles with minimal aggregates confirms that the protocol is successfully robust and reproducible with an efficiency for bulk quantity of around 100 mg Janus particles with little variation from one particle to another. Recently, Trihan et al. [46] have shown that hetero-aggregation between amine functionalized silica particles and citrate stabilized gold nanoparticles is efficient to optimize the total gold density and homogeneity, emphasising the particle size ratio of silica/gold to minimize the curvature effect. They obtained an isotropic surface coverage density of 15% with smaller AuNPs size (8 nm) on SiO_2_ particles of 600 nm. In our case, we attain a surface coverage density between 7% and 10% for ISO and Janus particles, a number in the same range of order of the one obtained by Perro et al. [22], from bulk synthesis with colloidosomes. From their TEM images, 72 AuNPs (of 15 nm) can be counted with a ~15 nm face-to-face distance on silica particles of 250 nm. With a different approach based on liquid/liquid interface in a microfluidic device, Abou-Hassan et al. synthesized Au–SiO_2_ Janus particles with a surface coverage density of ~10% [47].

In the literature, Janus particles have been highly reproduced from the Perro protocol based on a solidified emulsion pathway, with few characterisations on the Janus particles, however. Our originality here is to produce a family of particles with two different coverage rates (half and total surface) produced by a similar grafting process.

### 3.4. PTV Janus Particles Preparation

**Figure 6:**
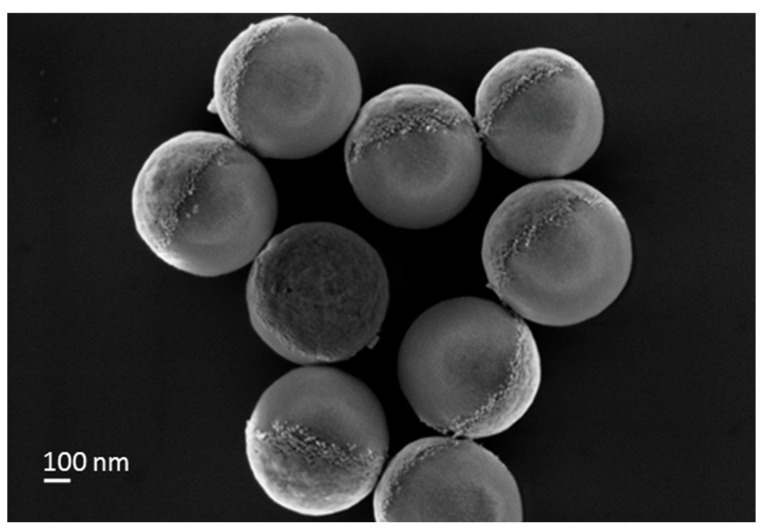
SEM images of PVD Janus gold functionalized SNP.

Another kind of Janus particle was produced by physical vapour deposition methods (PVD), gold on spin-coated SNPs on glass. The gold layer is deposited on a pre-layer of chromium to ensure good adhesion. A layer of 20 nm thickness gold was deposited, a value chosen to be close to the size of the gold NPs in the isotropic and Janus AuNPs/SNPs. Lastly, ultrasonication of the glass slide (submerged in Milli-Q^®^ water) ensured maximum extraction of physically produced gold/silica Janus particles (PVD). The PVD Janus particles were characterised by SEM (Figure 6).

Because of the sub-micron size of the particles, a focused ion beam (FIB) cannot be used for the characterisation of the gold layer, contrary to Rashidi’s studies on gold/polystyrene Janus particle of 5 μm diameter [48]. Therefore, rough estimation of the deposited gold layer was achieved by calculating the thickness of the gold layer from SEM observation of 17 PVD particles. At the resolution of ~5.6 nm/pixels, the value for the gold layer in central position is ~20.0 ± 2.0 nm, and ~18.0 ± 2.0 nm around the edges, which is due to the curvature of spherical silica particles. These values are in coherence with the targeted deposited value chosen for the physical vapour deposition.

### 3.5. Summary of the Different Class of Synthesized Particles

A family of four different particles have been synthesized. Table 2 lists the particles and their characteristic features (Janus or not) and briefly highlights the methods followed and the learning outcomes as important factors for their reproducibility. The Janus–PVD (PVD) particles are heavier by nearly a factor of two in comparison to Janus–AuNPs (JP).

### 3.6. Mobility in Bulk Solution

**Figure 7:**
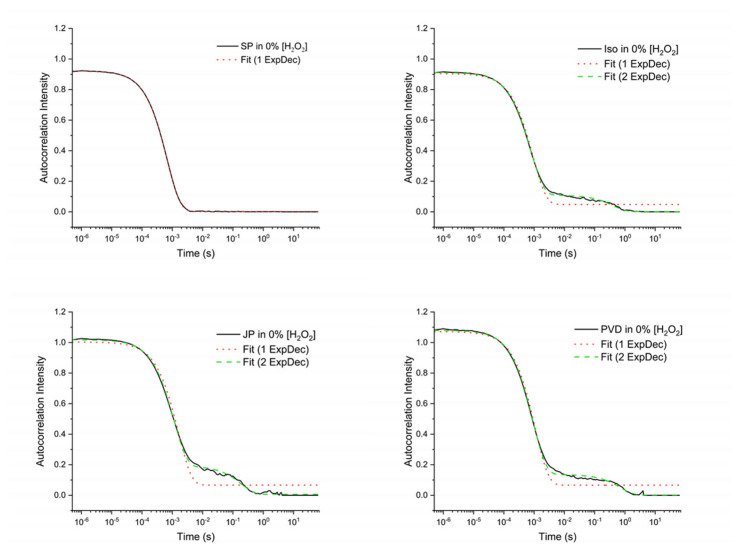
Curve fitting of respective DLS correlograms measured for the different particles (SNP, Iso, JP, and PVD) at 0%_v/v_ H_2_O_2_. Black solid lines represent the mean correlograms from DLS experiment. Red dotted lines represent curve-fit with single exponential decay function (“1 ExpDec”) and green dashed-lines represent curve-fit with double exponential decay function (“2 ExpDec”).

The mobility of the four types of particles was studied by dynamic light scattering in the presence or absence of H_2_O_2_. DLS has indeed the capability to characterize micro- and nanomotor motions. The correlation curves were fitted in most cases by one exponential, with a correlation time in the 7 × 10^−4^ s range. However, in some cases, a second exponential had to be added to adjust the signal, with a correlation time in the 3 × 10^−1^ s range (see Table S2 for the values and Figure 7 for a typical example of one and double exponential fitting).

These two exponential behaviours appear in the literature for particles having a significant degree of anisotropy. One DLS signal is coming from the translation motions and one from the rotational motion. For the size of particles considered, and the order of magnitude of the relaxation times that can be deduced from Equations (1) and (3), the fast signal can be assigned to translational motions and the slow one to rotational motions (when it is observed). However, the exact exploitation of the rotational component requires both polarized and depolarized DLS measurement that were not conducted here [35,49,50]. Therefore, only translational diffusion constants are presented in Table 3.

In the absence of H_2_O_2_, the diffusion coefficient can give access to the hydrodynamic radius through the Stokes–Einstein relationship (Equation (3)). The radii measured are very near the geometric ones expected for JP particles (see Table S3) which reached 700 nm! One explanation could be that the Janus particles present a unique heterogeneity of interfacial energy that is, in simulations, expected to impact their diffusion behaviour [51]. We may connect this specific interfacial energy to the colloidosomes process that might leave residual wax on the surface. Another explanation could be that the pronounced asymmetry of these particles could reinforce the rotational DLS signal for these samples, leading to an apparent enlargement of the translational signal.

In the presence of H_2_O_2_, we observed a significant evolution of the diffusion coefficient for ISO and JP. Their maximum increase in diffusion is approximately 30%, and is achieved at 1% in H_2_O_2_. These values are in agreement with other Janus systems composed of gold [49] and ten times lower than the one obtained on Pt. It is remarkable to observe an increase in diffusion for the isotropic particles in a homogeneous H_2_O_2_ environment. No significant variation was observed for SNPs, which was expected and PVD, which was more surprising.

### 3.7. Mobility in Microchannels

**Figure 8:**
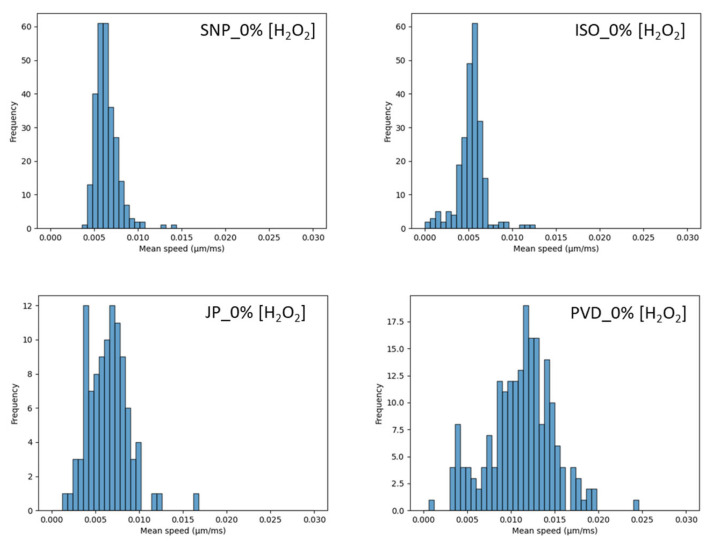
Mean speed of particles of the different particles without H_2_O_2_.

**Figure 9:**
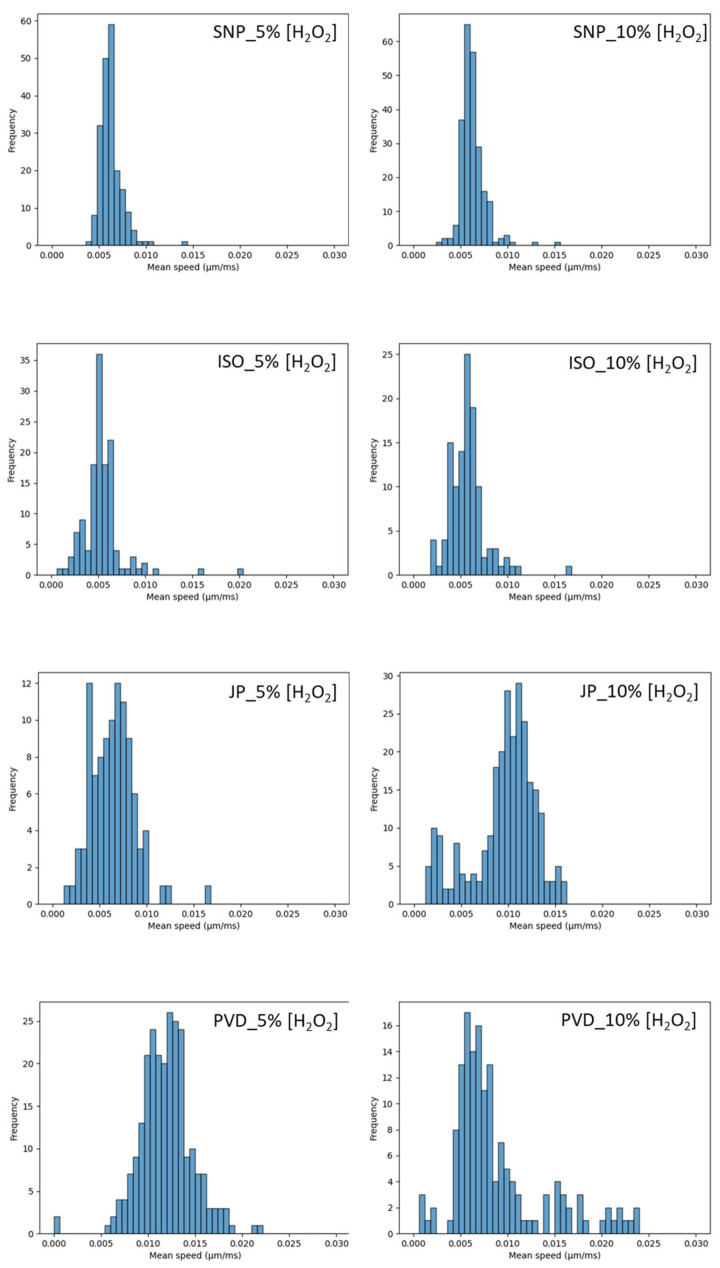
Mean speed of the different particles in the presence of 5 or 10%_v/v_ of H_2_O_2_.

A particle tracking velocimetry experiment (PTV) was developed in parallel to give access to a second method of diffusion measurement. These PTV measurements were conducted in microchannels (see Materials and Methods) in order to suppress any possible collective effects and Marangoni flows due to evaporation [52]. We must first notice that the uncertainties associated to these measurements are much higher than in DLS. This is due to known problems in sampling in PTV measurements [53] that might cover only a part of the tracking times available on DLS, which is the case in our study. Figure 8 and Figure 9 describe the speed distribution measured for the particles without H_2_O_2_, and with 5% or 10% of H_2_O_2_, respectively. For SNPs, the speed distribution shape is close to a log-normal distribution. It is not modified by H_2_O_2_ and could reveal a log-normal size distribution of the silica particles. For ISO, at 0%_v/v_ H_2_O_2_, the majority of the particles are concentrated around a mean value and could be described by a Gaussian speed distribution. However, a residual few number of particles are distributed on a large speed range, traducing heterogeneities in the sample. With increasing H_2_O_2_, the distribution is enlarged with an increase of the average value. The decrease of some particle speed in comparison to SNPs could be related to the increase weight of some article, by aggregation for example. For Janus and PVP, at 0%_v/v_ of H_2_O_2_, the speed distribution is larger, with even two visible distributions for PVD case, revealing some heterogeneities in the sample (interaction of particles with channel edge, aggregates, interaction between particles, etc…). There is a clear acceleration of PVD particles in comparison to others in the absence of H_2_O_2_. In the presence of H_2_O_2_, the average speed of particles increases with H_2_O_2_ content for both JP and PVD. For the 10%_v/v_ case, JP and PVD particles exhibit a major peak distribution with few particles distributed on a large speed range value. The average speed value is extracted from the major peak. Table 4 summarizes the average values of the distributions. To compare the PTV analysis to DLS ones, we extract the mean diffusion coefficients from the mean speed values of Figure 8 and Figure 9 according to Equation (7) and then extract the average diffusion coefficient (Table 5).

If we consider now only the averaged values of the diffusion coefficient in the absence of H_2_O_2_, the values for SNP, ISO, and JP are quite similar in DLS and PTV. However, the one for PVD are too far from the expected range to have a physical meaning. Due to the acquisition time used for the PTV (~100 ms), we may observe here gravitational effects due to the large weight of the particles (see Table 2) [54]. Sedimentation induces a mixing effect with an artificial increase of diffusion coefficient due to the renewal of H_2_O_2_ reactant.

In the presence of H_2_O_2_, the increase on ISO and JP diffusion (20% to 30%) is also compatible with the one measured by DLS in the 1%_v/v_ to 5%_v/v_ H_2_O_2_ range. However, JP appear much more efficient at very high H_2_O_2_ concentrations, reaching 110% increase in diffusion.

These results demonstrate the need for a multi-technique approach to account for the different bias of each technique: PTV dealing with single particles presenting individual behaviour and a strong need for statistic; but that is capable to reveal heterogeneities of the sample, DLS that can be plagued by aggregation or rotational components.

### 3.8. Comparison

Our approach, based on a common synthetic platform, first revealed bias in the values measured in both methods used in particle motion qualification for Janus particles, DLS and PTV. This strongly supports the need for a comparison of data measured with the two techniques. For both techniques, a variation in diffusion coefficient is visible for gold functionalized SNPs particles in the presence of H_2_O_2_, ensuring an effect of the reactivity between gold and H_2_O_2_. The chosen approach also demonstrated that the explicit preparation of Janus structure is not mandatory to obtain a motion at intermediate fuel concentration. In Table 5, it is indeed shown that a Janus particle is only marginally faster than the so-called ISO ones. However, if isotropic particles can achieve motion in chemical gradients, asymmetry is theoretically mandatory for homogeneous fuel concentration [16]. We can therefore consider three different possible hypotheses to explain the motions:

(1) The catalytic activity creates local gradient due to insufficient mixing during the measurement. However, it cannot be the case in microfluidic experiments as particles explore all the space of the channels.
(2) The catalytic activity is sufficiently low to occur at the same time only on a limited number of spots on the particle. The low number of spots distributed randomly will thus create an asymmetry. If it may not be sufficient for the production of a local chemical gradient, this may lead to asymmetric local heating, and thus to thermophoresis. This may explain why ISO and JP have similar efficiency at intermediate concentrations in fuel (<5%), but not at high concentration (10%_v/v_) where the reactive sites will be more synchronous.
(3) AuNPs are not distributed evenly enough on the silica surface to ensure a real isotropy. This hypothesis was indeed introduced to explain the chemotaxis of PLGA gold particles [14]. The main argument for this is the observation of a rotational component in ISO DLS signal that is the signature of anisotropy. The defects in the organization of AuNPs on ISO particle are sufficient to provide them with their motion capabilities.

Our result does not allow us to distinguish between the two latter explanations and may be a combination of the two phenomena.

## 4. Conclusions

A family of different silica particles functionalized by gold were synthesized to compare their mobility in the presence of H_2_O_2_. A comparison of Janus and isotropic particles is rare in the literature; a comparison could help to optimize particles with higher efficacy. The design of the particles was chosen (1) to ensure similar grafting of gold nanoparticles for isotropic and Janus configuration, and (2) to produce Janus particles with two different gold functionalities (gold nanoparticles and a gold layer whose thickness is equivalent to the AuNPs diameter ~20 nm). Mobility was compared through DLS (3D in batch) and PTV analysis (in a microfluidic channel to avoid collective effect). Whatever the functionalization, the particles are mobile in the presence of H_2_O_2_. If analysis revealed bias in the values measured in both methods (DLS and PTV), a general trend can be observed showing that, for intermediate fuel concentration, a Janus structure is not mandatory to obtain a motion. The mechanism at work needs further investigation, but it is open to the promising possibility for the design of self-propelled particles.

## Figures and Tables

**Table 1:** Organisation of the AuNPs functionalized SNPs. AuNPs/SNP is the number of AuNPs per silica particles counted from visible part in SEM images and d_cc_ the average distance between the centres of 2 AuNPs on silica particles measured when particles are aligned or in average from the counted AuNPs on SiO_2_ particles.

Particles	AuNPs/SNP	Au–Au Centre to Centre Distance (d_cc_ in nm) (*)
SiO_2__AuNPS_iso (ISO)	220–280	~35 to 40 nm
SiO_2__AuNPS_Janus (JP)	110–150	~35 to 40 nm

(*) The batch used for analysis was prepared with AuNPs size of ~20 nm.

**Table 2:** Summary of key factors in the methods followed for synthesizing different particles. The particle size was calculated with respect to an averaged gold thickness on their surface for comparison purpose.

Type of Particle, Averaged Particle Size and Weight	Method	Important Factors for Reproducibility. Reproducibility Achieved	Maximal Amount That Can Be Prepared Time for Preparation
**AuNPs** **25 nm, 1.6 × 10^−16^ g**	Reverse Turkevich	Gold/citrate ratio, temperature, citrate concentration. ±5 nm from batch to batch	10 mL or 50 mL of AuNPs suspension at 0.25 mM in Au per batch Time for preparation ~1 h (after 1 day of rest)
**SNP** **475 nm, 1.2 × 10^−13^ g**	Stöber	TEOS/EtOH/H_2_O ratios and Precursors Mixing Rate.	30 mL of silica NPs (475 nm) suspension at 30 mg/mL in SiO_2_ i.e., 2.4 × 10^11^ part/mL. **(~900 mg of SiO_2_ NPs per batch)** Time for preparation ~4 H
**Iso** **480 nm 1.6 × 10^−13^ g**	Surface functionalization	APTES Catalysis and AuNPs concentration. Interaction time (for APTES and for AuNPs) Colloidal stability of the SNPs Number of AuNPs grafted particles per SNP: 220–280	2 steps: functionalization by NH_2_ and then AuNPs grafting. 5 mL of Iso at 1.22 × 10^10^ part/mL **~10 mg of Iso from 7.5 mg of SNPs** **Easy scale-up** Time for preparation: 1.5 day
**JP** **478 nm 1.4 × 10^−13^ g**	Pickering Emulsion and surface functionalization	Molar ratio of SNP:CTAB and mass ratio of SNP:Wax:water (for Pickering emulsion and Colloidosome preparation), mass ratio of SNP:APTES (for SNP functionalization with APTES) and mass ratio of SNP_NH2:AuNPs (for APTES-SNP functionalization with AuNPs) Solvent composition for APTES grafting Emulsification energy (for Pickering Emulsion) Quenching of emulsion Solvent for Wax removal Number of AuNPs grafted particles: 110–150	Based on colloidosome preparation. ~2 to 3 g of colloidosomes per batch. Time for preparation: 2 days 0.5 g of colloidosomes used to obtain at the end 5 mL of Janus-AuNPs at ~1.6 × 10^13^ part/mL **(~20 mg of JP from 0.5 g of colloidosomes**). Easy scale-up Time of preparation:1.5 day
**JP_PVD** **485 nm 2.7 × 10^−13^ g**	Physical Vapor Deposition	Monolayer assembly of SNPs on substrate Intermediate adhesion layer between Gold and SNPs Thickness of gold layer: 20 nm ± 1 nm	~1.7 × 10^10^ particles/batch (from a 37.5 cm^2^ glass coating) Ie 1 mL of suspension at 1.7 × 10^10^ particles/mL **(~4.4 mg per batch**) No easy scale-up Time of preparation: 2 H

**Table 3:** Translational diffusion coefficient obtained from the DLS correlograms analysis for different H_2_O_2_ concentration. Higher concentrations could not be measured owing to the formation of bubbles.

%_v/v_ [H_2_O_2_]	Diffusion Coefficient (µm^2^/s) ± Std. Dev			
	SNP	Iso	JP	PVD
**0**	1.05 ± 0.02	1.03 ± 0.05	0.70 ± 0.06	0.86 ± 0.05
**0.5**	1.07 ± 0.01	1.25 ± 0.08	0.80 ± 0.06	0.86 ± 0.06
**1**	1.05 ± 0.01	1.32 ± 0.16	0.88 ± 0.13	0.90 ± 0.05
**3**	1.05 ± 0.02	1.22 ± 0.01	0.89 ± 0.08	0.86 ± 0.02
**5**	1.06 ± 0.02	1.23 ± 0.02	0.90 ± 0.04	0.85 ± 0.03

**Table 4:** Mean speed obtained from PTV analysis for different H_2_O_2_ concentration. In comparison to Figure 8 and Figure 9, the Mean speed was transformed into µm/s.

%_v/v_ [H_2_O_2_]	Mean Speed (µm/ms) ± Std Dev			
	SNP	Iso	JP	PVD
**0**	6.4 ± 1.3	5.3 ± 1.5	6.4 ± 2.3	11.1 ± 3.7
**5**	6.2 ± 1.1	5.4 ± 2.2	7.5 ± 2.1	11.9 ± 2.8
**10**	6.2 ± 1.2	5.6 ± 1.9	9.1 ± 3.6	8.9 ± 4.5

**Table 5:** Average diffusion coefficient obtained from the mean speed values issued from PTV analysis for different H_2_O_2_ concentration. The diffusion coefficient and the standard deviation are calculated from the data in the 90^th^ percentile.

%_v/v_ [H_2_O_2_]	Average Diffusion Coefficient (μm^2^/s), (range)			
	SNP	Iso	JP	PVD
**0**	0.87± 0.22	0.65 ± 0.18	0.93 ± 0.53	2.6 ± 0.98
**5**	0.84 ± 0.16	0.68 ± 0.23	1.30 ± 0.36	3.07 ± 0.8
**10**	0.84 ± 0.18	0.76 ± 0.24	2.36 ± 1.12	1.41 ± 0.83

## Data Availability

Data is contained within the article.

## Supplementary Materials

The following supporting information can be downloaded at: https://www.mdpi.com/article/10.3390/nano14221796/s1, S1. Silica particles, synthesis and characterization, S2. AuNPs synthesis, S3. Colloidosomes, S4. Surface functionalization of silica nanoparticles, S5. SNP with grafted AuNPs, S6. DLS analysis and extraction of correlation time. Including: Figure S1: Monolayer of SNP from spin-coating on plasma treated wafer., Figure S2: DLS analysis of SiO_2_ NPs in suspension in water. (left) correlogram (right) size distribution., Figure S3: SEM images of SNP at 10K x magnification showing SNP after 60 min ultrasonic bath showing no structural damage., Figure S4: DLS analysis of AuNPs suspension in water, synthesized from reverse Turkevich protocol. (left) correloram (right) size distribution., Figure S5: Zeta potential versus CTAB concentration at t = 30 min after addition of CTAB in SNP suspension., Figure S6: SEM images of the colloidosomes synthesized with different CTAB concentrations: (a) 0.004 mM, (b) 0.02 mM, (c) 0.04mM, (d) 0.08 mM., Figure S7: SEM images of two colloidosomes synthesized with a CTAB concentration of 0.004mM., Figure S8: SEM image of a colloidosome (on the left) and its surface (on the right), synthesized with a CTAB concentration of 0.08M., Figure S9: Mean size calculated from 60 Colloidosomes at respective concentration of CTAB., Figure S10: SEM image of SNPs-NH2 after the dissolution of the wax in hexane., Figure S11: SEM-EDX image and Cartography result of ISO sample (A and B respectively). Cartography image and elemental spectra map (C) confirming the presence of gold nanoparticles (Au) in Isotropic gold/silica particle., Figure S12: SEM-EDX image and Cartography result for chemically synthesized Gold/Silica Janus particle (JP) sample (A and B respectively). Cartography image (B) and elemental spectra map (C) confirms the presence of gold nanoparticles (Au) in Janus particles., Table S1: Colloidosome mean size at respective CTAB concentration., Table S2: Correlation times issued from one or double exponential fitting of the correlograms measured by DLS for the different particles families., Table S3: Diameter calculated from the Diffusion coefficient.
