# Supplementary material for: Making Mobile Nanotechnology Accessible: Is the Explicit Preparation of Janus Nanoparticle Necessary to Achieve Mobility?
Source: Nanomaterials (Basel). 2024 Nov 8;14(22):1796. doi: 10.3390/nano14221796 (PMC11597384; doi:10.3390/nano14221796)
Supplement: Supplementary file 1 [file nanomaterials-14-01796-s001.zip › nanomaterials-3225861-supplementary.pdf]

Supplementary information

Making Mobile Nanotechnology Accessible: Is the Explicit Preparation of Janus Nanoparticle Necessary to Achieve Mobility?

Vagisha Nidhi, Arthur Allaire, Zakariya Ait Athmane, Patrick Guenoun, Fabienne Testard *, Jean-Philippe Renault * and Florent Malloggi *

Université Paris-Saclay, CEA Saclay, CNRS, NIMBE, UMR 3685, LIONS, 91191 Gif-Sur-Yvette CEDEX, France; vagisha.nidhi@gmail.com (V.N.); arthur.allaire@ens.psl.eu (A.A.); zakariya.aitathmane@cea.fr (Z.A.A.); patrick.guenoun@cea.fr (P.G.)

* Correspondence: fabienne.testard@cea.fr (F.T.); jean-philippe.renault@cea.fr (J.-P.R.); florent.malloggi@cea.fr (F.M.); Tel.: +33-169-081-550 (J.-P.R.)

S1. Silica particles, synthesis and characterization

The silica particles are synthesized following the stöber method [1], using the conditions described in Marta Ibisate Muñoz phD [2] where the required amount of water/NH3 and TEOS to be added in ethanol are tabulated in function of the desired size of silica particles. Typically, water, ammonia and ethanol are added successively to a volume of ethanol. The mixture was then stirred for 2h. For example, for an aimed diameter about 480 nm, the concentrations of water/ammonia/TEOS in the full mixture containing ethanol are 24 M /1.5 M /0.25 M. [2]

After the reaction, washing and concentration is performed through centrifugation cycle. Precisely, the solution is centrifuged at 7500 rpm for 15 min with 50 mL polypropylene tubes, using a sigma rotor (Nr. 12156-H, 8x140g, max. 16500/min). After removing the supernatant, the pellet is resuspended in ≈ 40 mL of absolute ethanol under 15 mn of sonication before repeating the centrifugation cycle. The procedure is repeated two times, to finally disperse the solution in 30 ml of ethanol for storage in the fridge (~30 mg/ml of SiO_2). For each synthesized batch of silica particles (called SNP), the approximate concentration of silica is extracted by weight after drying, the size of silica particles is measured by SEM on 50 particles and hydrodynamic diameter analyzed by DLS. For example for the synthetic condition mentioned, the measured diameter was of 475nm

The Figure S1 and SI 2 shows SEM and DLS of a typical batch of silica particles synthesized [1].

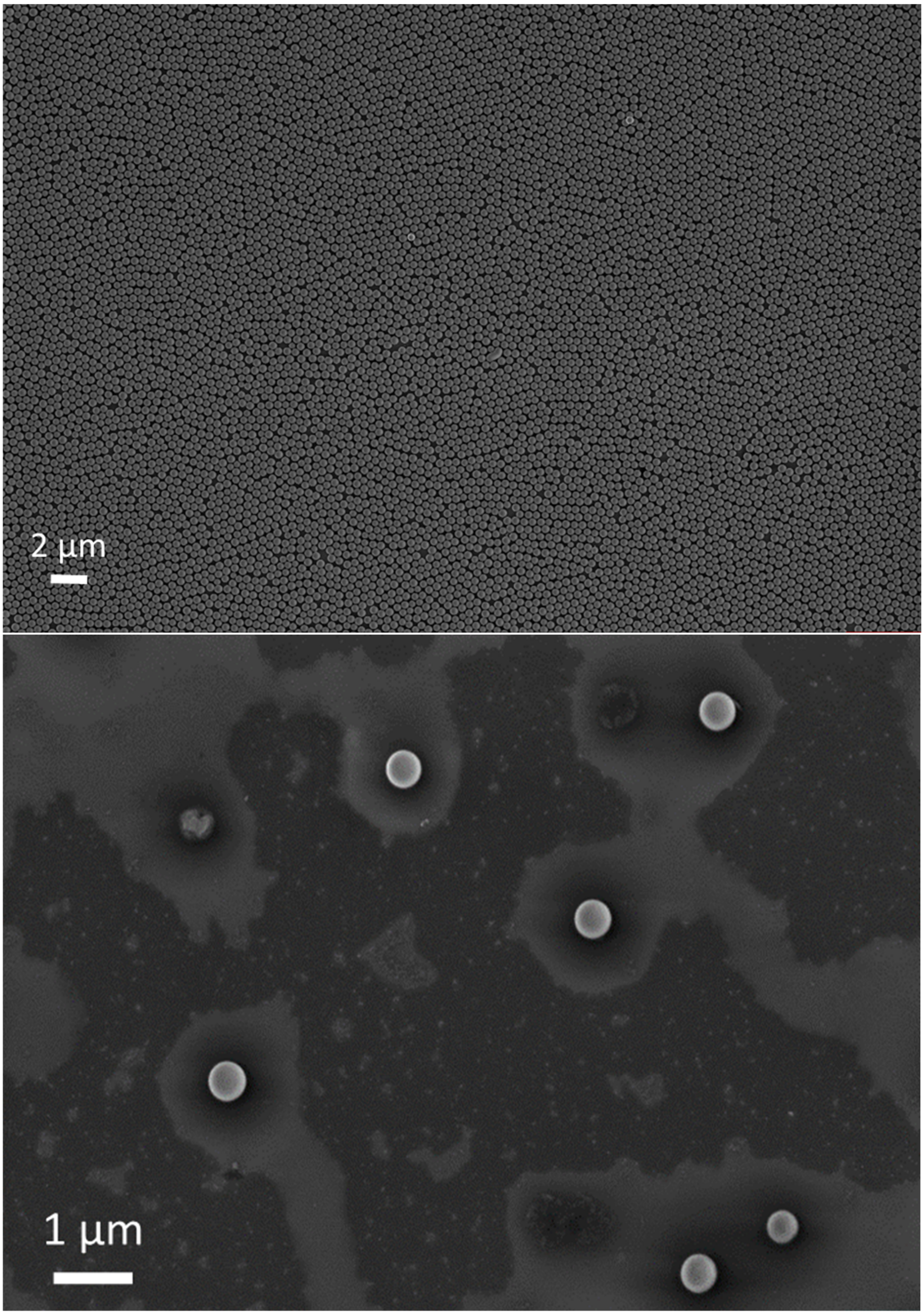

Figure S1 : Monolayer of SNP from spin-coating on plasma treated wafer.

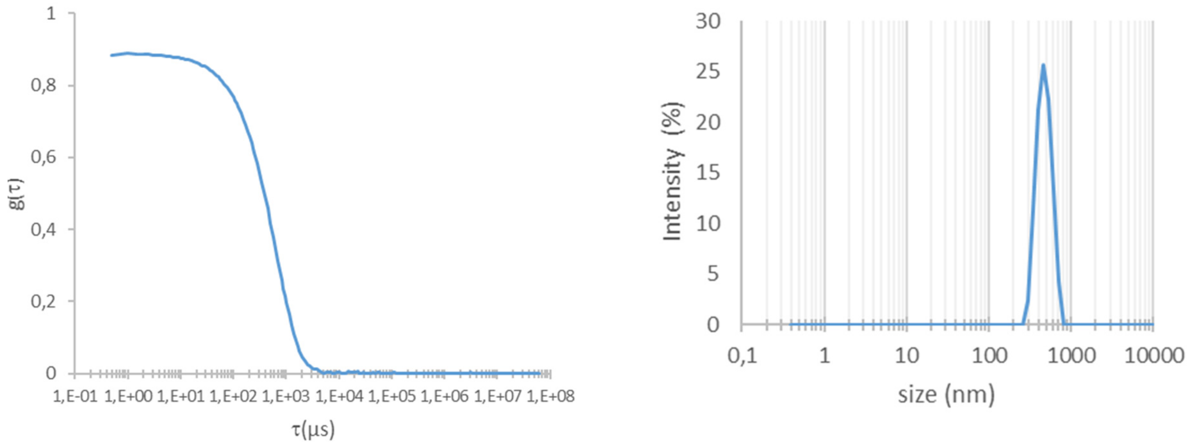

Figure S2 : DLS analysis of SiO2 NPs in suspension in water. (left) correlogram (right) size distribution

SEM analysis of SIO_2 particles after a cycle of 60 mn in a ultrasonic bath shows that the particles are

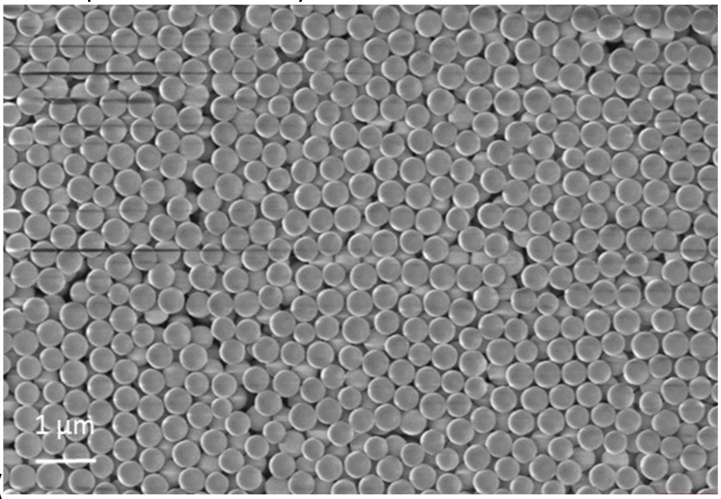

not modified (

Figure *S3*).

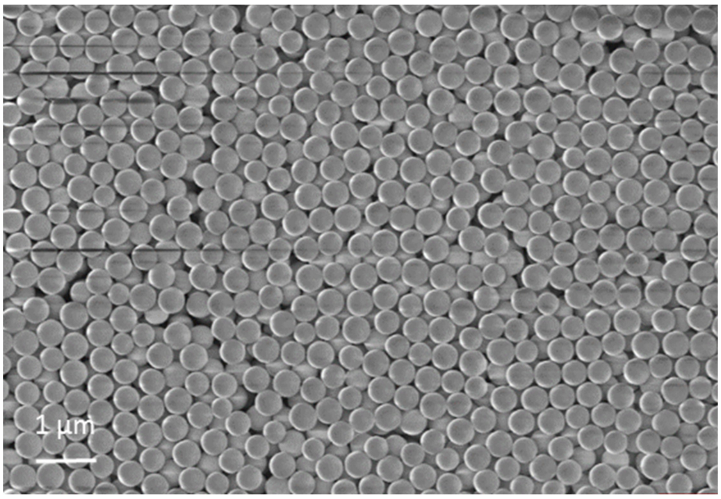

Figure S3 : SEM images of SNP at 10K x magnification showing SNP after 60 min ultrasonic bath showing no structural damage.

S2. AuNPs synthesis

The procedure is adapted from the one described by S.K. Sivaraman et al [3]. Prior to the synthesis, all glassware were washed using aqua regia and rinsed with water. In an Erlenmeyer, 2,00 mL of a sodium citrate solution (C=34 mM) prepared in citric acid (0.05% w/v) were added to 47,75 mL of boiling water. 250 µL from a stock solution of $HAuCl_4{,}3H_2O$ (C= 0,05M) were added in the solution to attain a citrate to chloroauric molar ratio MR equal to 5.44. It was kept boiling for 2 min before the solution is let covered by an aluminium foil to cool down at room temperature for 24h before characterization. The hydrodynamic radius of the as produced nanoparticles (called Au-NPs) measured by DLS varied from batch to batch from 21 nm to 28 nm (see Fig. SI-4 for a batch at 22nm) with PDI as low as 0.09. The suspension is kept in the fridge before use.

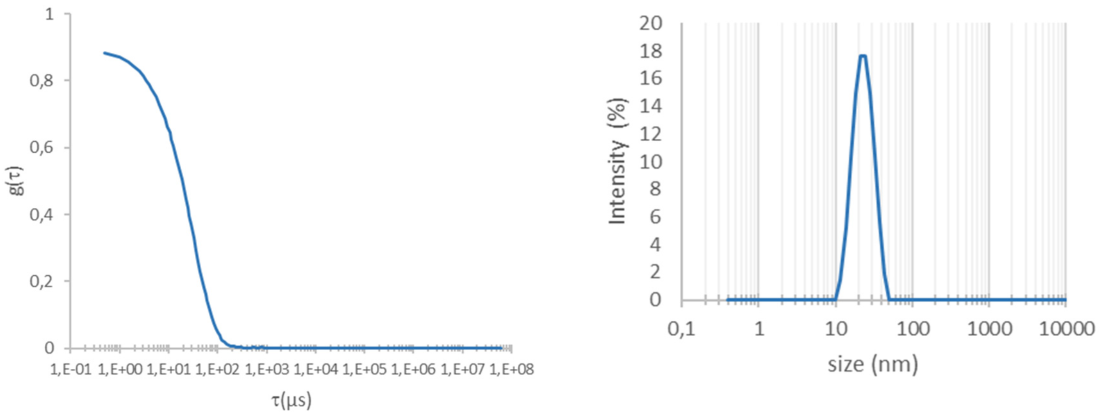

Figure S4 : DLS analysis of AuNPs suspension in water, synthesized from reverse Turkevich protocol. (left) correloram (right) size distribution.

S3. Colloidosomes

To find the CTAB optimal concentration to procude colloidosomes with waxs and SiO2 NPs of 475 nm size, the Zeta potential of silica particles (SNP) in water has been measured in function of the concentration of a cationic surfactant the cetyltrimethylammonium bromide (CTAB) at t=30 mn after the addition of the CTAB (Figure S5). In addition, the obtained colloidosomes have been characterized by SEM (Fig. SI 6, 7 and 8).

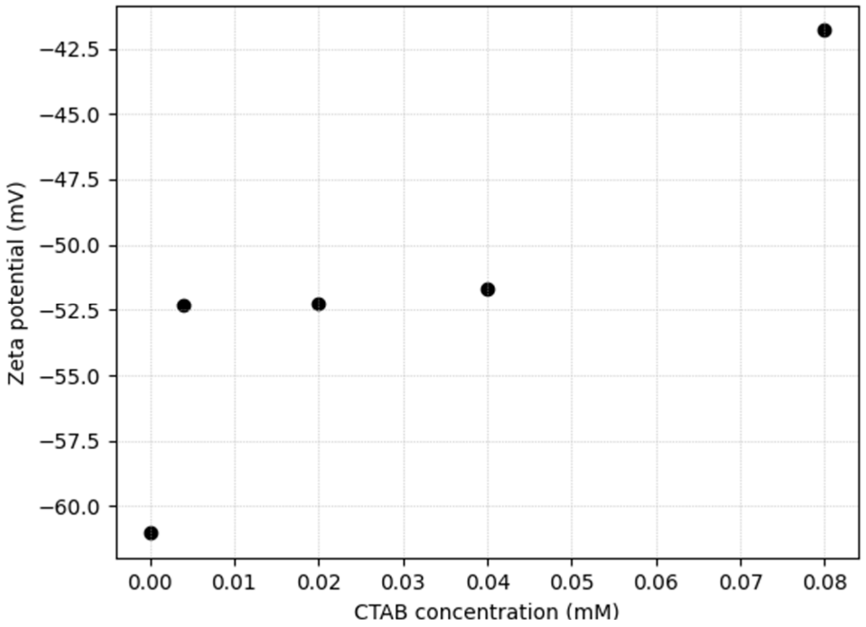

Figure S5 : Zeta potential versus CTAB concentration at t= 30 min after addition of CTAB in SNP suspension.

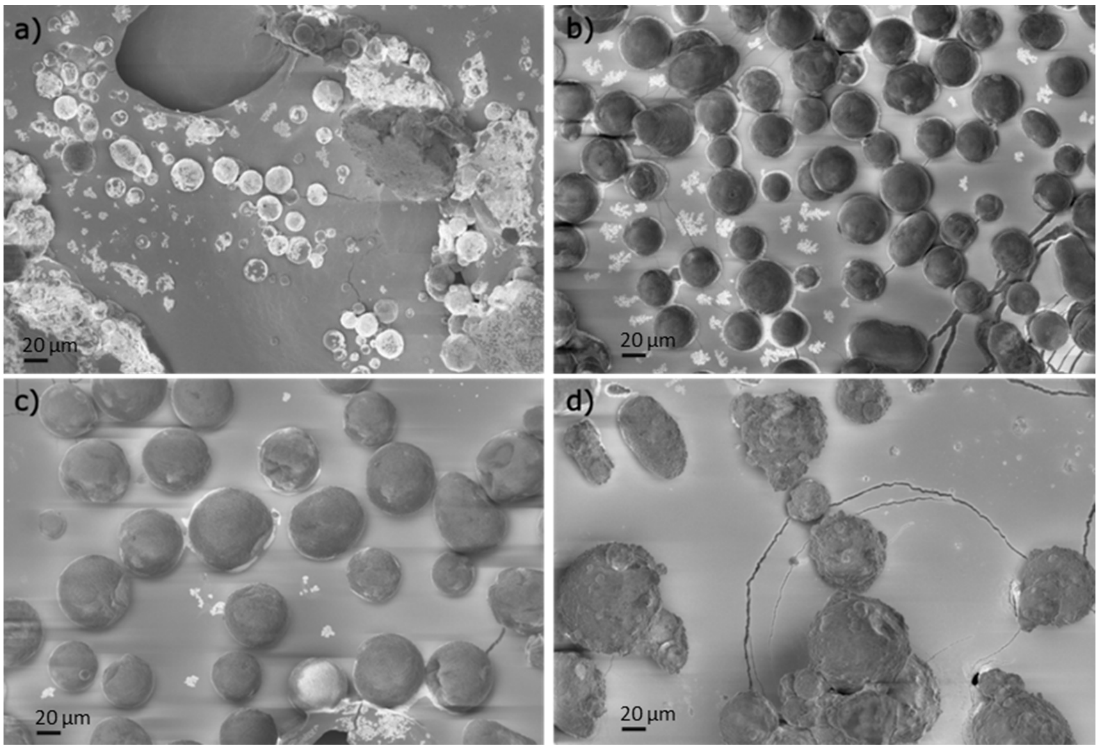

Figure S6 : SEM images of the colloidosomes synthesized with different CTAB concentrations: a) 0.004 mM, b) 0.02 mM, c) 0.04mM, d) 0.08 mM.

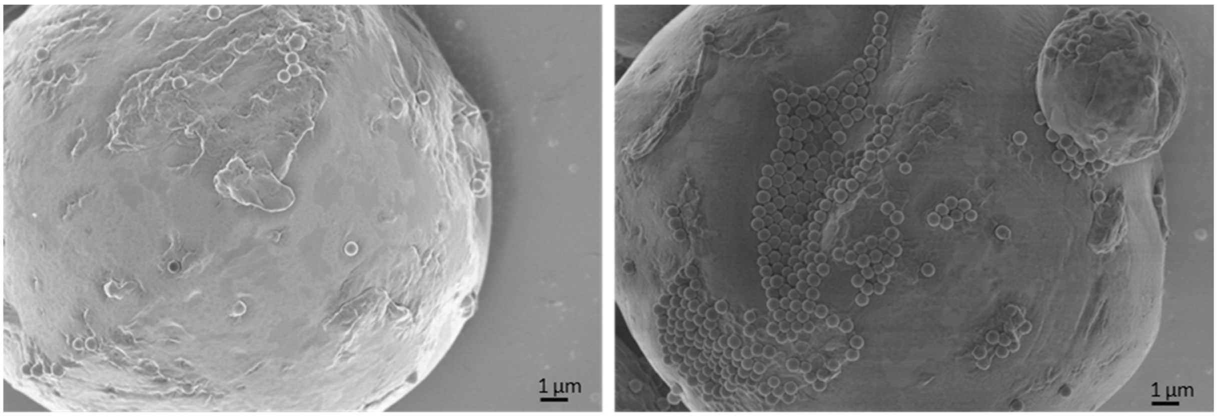

Figure S7 : SEM images of two colloidosomes synthesized with a CTAB concentration of 0.004mM.

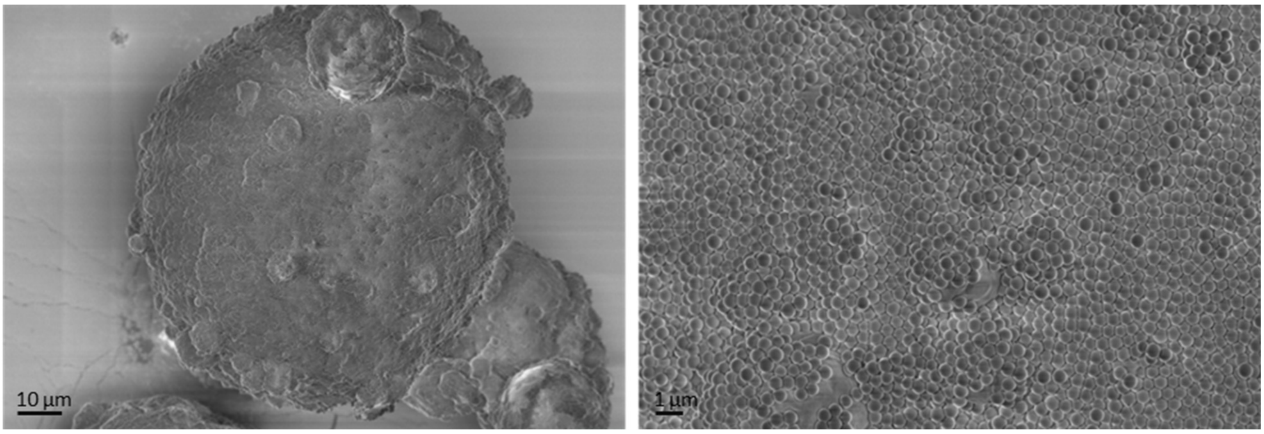

Figure S8 : SEM image of a colloidosome (on the left) and its surface (on the right), synthesized with a CTAB concentration of 0.08M

The size of the colloidosomes was measured from SEM analysis and summarized in Figure S9. The evolution is opposite to the tendency observed by Avossa et al [4] but the particle size, the nature of the wax and the energy mixing are different in our study.

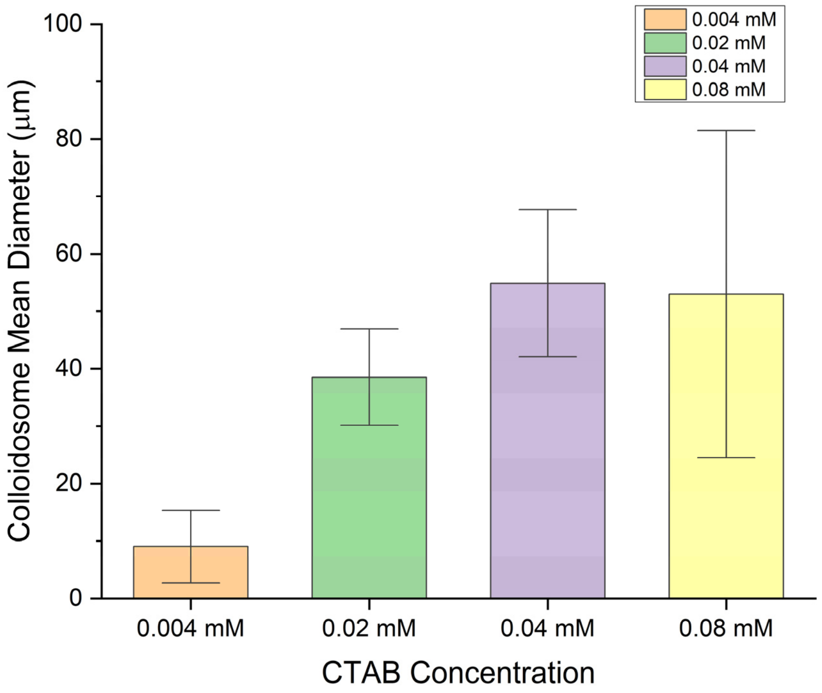

Figure S9 : Mean size calculated from 60 Colloidosomes at respective concentration of CTAB

Table S1 : Colloidosome mean size at respective CTAB concentration

CTAB (mM)	Colloidosome Mean Diameter (µm) ± SD
0.004	9.1 ± 6.3
0.02	38.5 ± 8.4
0.04	54.9 ± 12.8
0.08	53.0 ± 28.5

S4. Surface functionalization of silica nanoparticles

The silica particles embedded at the surface of the colloidosomes are functionalized on the outer part by APTES and recovered in a second step after dissolving the wax in hexane (Figure S10) shows that no residual wax is observed on the functionalized silica particles.

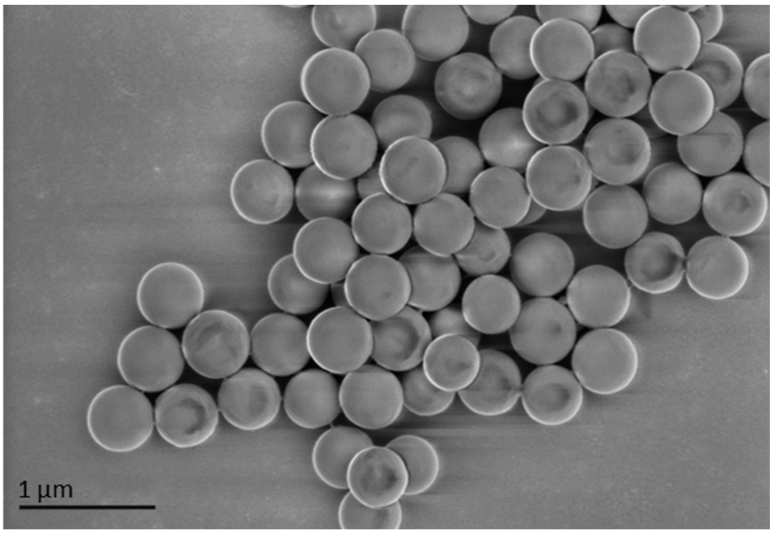

Figure S10 : SEM image of SNPs-NH2 after the dissolution of the wax in hexane

S5. SNP with grafted AuNPs

The isotrope and Janus particles functionalized by AuNPs have been caraterized by SEM-EDX respectively. The high intensity for Si comes from the sample wafer used for analysis. The presence of gold is confirmed by the analysis.

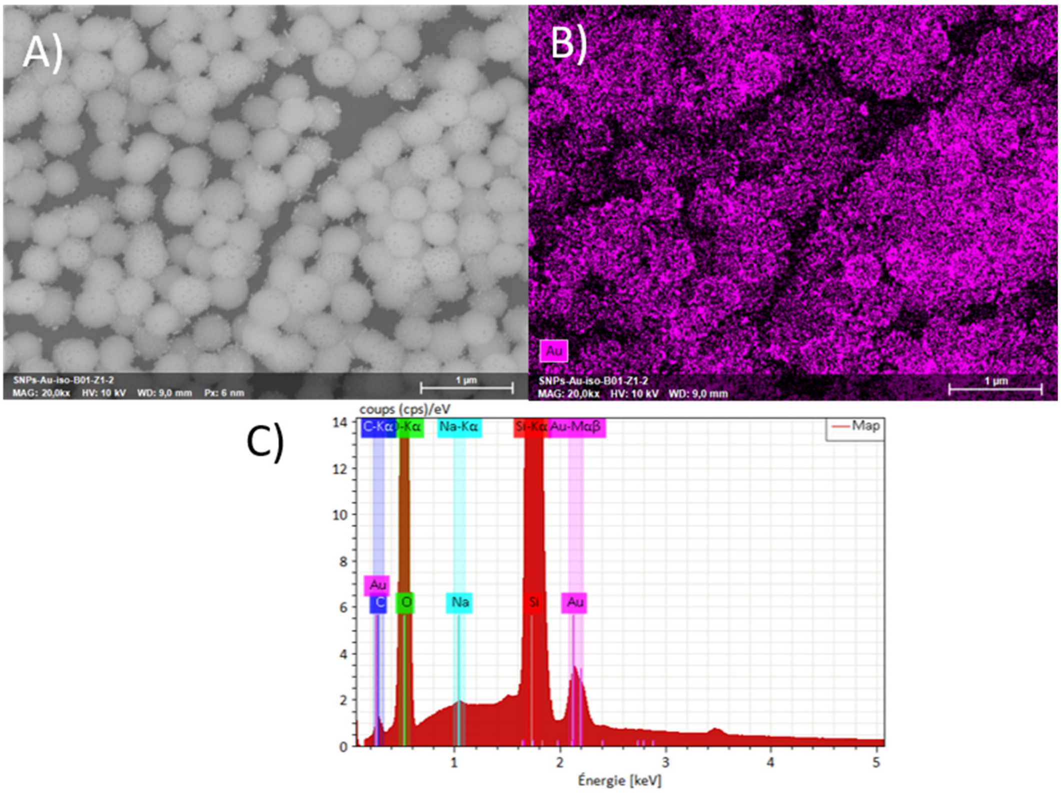

Figure S11 : SEM-EDX image and Cartography result of ISO sample (A and B respectively). Cartography image and elemental spectra map (C) confirming the presence of gold nanoparticles (Au) in Isotropic gold/silica particle.

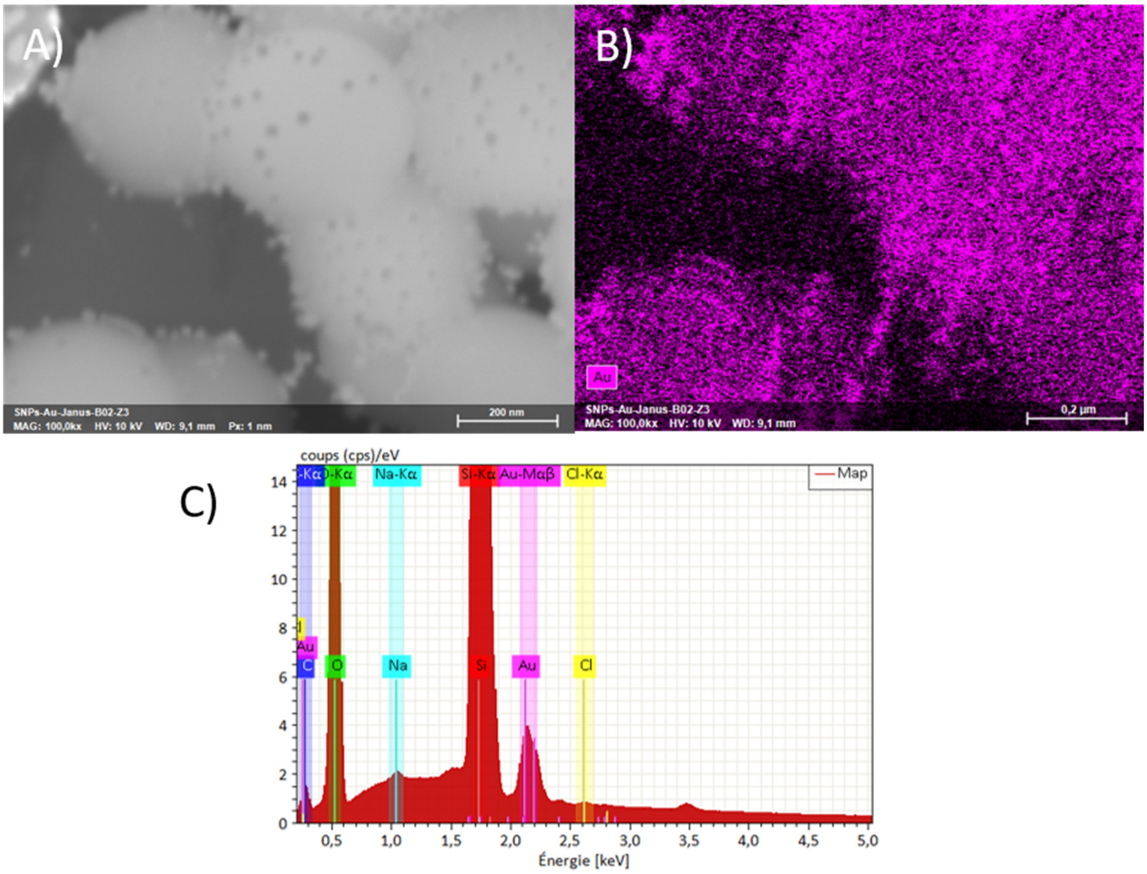

Figure S12 : SEM-EDX image and Cartography result for chemically synthesized Gold/Silica Janus particle (JP) sample (A and B respectively). Cartography image (B) and elemental spectra map (C) confirms the presence of gold nanoparticles (Au) in Janus particles.

S6. DLS analysis and extraction of correlation time

The correlograms can be fitted with one exponential characterized by the correlation time T_1(s), but for sometimes a second contribution should be added with the fit by a second exponential characterized by the second correlation time T_2(s).

The Table S2 summarized the correlation times found for the different particles family in function of the H_2O_2 concentration expressed in $\%_{v/v}$.

Table S2 : Correlation times issued from one or double exponential fitting of the correlograms measured by DLS for the different particles families.

**% (v/v) [H_2O_2]**	**Correlation time T1(s) and T2(s)**						
	**SP**	**Iso**		**JP**		**JP-PVD**	
	T_1 (s)	T_1 (s)	T_2 (s)	T_1 (s)	T_2 (s)	T_1 (s)	T_2 (s)
0	$6.90\ 10^{-4}$	$7.07\ 10^{-4}$	$3.54\ 10^{-1}$	$1.04\ 10^{-3}$	$4.05\ 10^{-1}$	$8.41\ 10^{-4}$	$2.78\ 10^{-1}$
0.5	$6.76\ 10^{-4}$	$5.83\ 10^{-4}$	n.d.	$9.03\ 10^{-4}$	$2.53\ 10^{-1}$	$8.43\ 10^{-4}$	n.d.
1.0	$6.91\ 10^{-4}$	$5.53\ 10^{-4}$	n.d.	$8.34\ 10^{-4}$	n.d.	$8.08\ 10^{-4}$	n.d.
3.0	$6.92\ 10^{-4}$	5.9610^{-4}	$2.13\ 10^{-1}$	$8.17\ 10^{-4}$	n.d.	$8.44\ 10^{-4}$	n.d.
5.0	$6.88\ 10^{-4}$	$5.92\ 10^{-4}$	$1.85\ 10^{-1}$	$8.11\ 10^{-4}$	$2.53\ 10^{-1}$	$8.51\ 10^{-4}$	$2.15\ 10^{-1}$

The expected relaxation time for a 475 nm particle size calculated from Stokes equation for rotational a Brownian motion is 0.054 s^{-1}.

The hydrodynamic diameter issued from the Diffusion coefficient through Stokes Einstein relationship are presented in Table S3.

Table S3 : Diameter calculated from the Diffusion coefficient

% (v/v) [H_2O_2]	Diameter (nm) ± Std. Dev (%)			
	**SP**	**Iso**	**JP**	**JP-PVD**
0	467 ± 2.2	478 ± 5.5	705 ± 8.9	569 ± 6.2
0.5	457 ± 1.2	394 ± 5.9	610 ± 7.3	570 ± 6.6
1	467 ± 1.4	374 ± 11.3	564 ± 12.4	547 ± 5.1
3	468 ± 2.1	403 ± 1	553 ± 8.6	571 ± 3.0
5	465 ± 2.4	400 ± 2.1	549 ± 5.1	576 ± 4.3

S7. Bibliography:

1. Stöber, W.; Fink, A.; Bohn, E. Controlled Growth of Monodisperse Silica Spheres in the Micron Size Range. *J. Colloid Interface Sci.* **1968**, *26*, 62–69, doi:10.1016/0021-9797(68)90272-5.
2. Muñoz, M.I. CRISTALES FOTÓNICOS BASADOS EN ÓPALOS. phD, Universidad Autónoma de Madrid, 2003.
3. Sivaraman, S.K.; Kumar, S.; Santhanam, V. Monodisperse Sub-10 Nm Gold Nanoparticles by Reversing the Order of Addition in Turkevich Method–the Role of Chloroauric Acid. *J. Colloid Interface Sci.* **2011**, *361*, 543–547, doi:10.1016/j.jcis.2011.06.015.
4. Avossa, J.; Esteves, A.C.C. Influence of Experimental Parameters on the Formation and Stability of Silica-Wax Colloidosomes. *J. Colloid Interface Sci.* **2020**, *561*, 244–256, doi:10.1016/j.jcis.2019.11.011.
